# Inhibition of A_2A_ Adenosine Receptor Signaling in Cancer Cells Proliferation by the Novel Antagonist TP455

**DOI:** 10.3389/fphar.2017.00888

**Published:** 2017-12-01

**Authors:** Stefania Gessi, Serena Bencivenni, Enrica Battistello, Fabrizio Vincenzi, Vittoria Colotta, Daniela Catarzi, Flavia Varano, Stefania Merighi, Pier Andrea Borea, Katia Varani

**Affiliations:** ^1^Department of Medical Sciences, Pharmacology Section, University of Ferrara, Ferrara, Italy; ^2^Department of Neuroscience, Psychology, Drug Research and Child Health, Pharmaceutical and Nutraceutical Section, University of Florence, Florence, Italy

**Keywords:** A_2A_ adenosine receptor, cancer cell proliferation, intracellular signaling pathways, Drug Discovery and Therapy, receptor antagonist

## Abstract

Several evidences indicate that the ubiquitous nucleoside adenosine, acting through A_1_, A_2A_, A_2B_, and A_3_ receptor (AR) subtypes, plays crucial roles in tumor development. Adenosine has contrasting effects on cell proliferation depending on the engagement of different receptor subtypes in various tumors. The involvement of A_2A_ARs in human A375 melanoma, as well as in human A549 lung and rat MRMT1 breast carcinoma proliferation has been evaluated in view of the availability of a novel A_2A_AR antagonist, with high affinity and selectivity, named as 2-(2-furanyl)-N^5^-(2-methoxybenzyl)[1,3]thiazolo[5,4-d]pyrimidine-5,7-diammine (TP455). Specifically, the signaling pathways triggered in the cancer cells of different origin and the antagonist effect of TP455 were investigated. The A_2A_AR protein expression was evaluated through receptor binding assays. Furthermore, the effect of A_2A_AR activation on cell proliferation at 24, 48 and 72 hours was studied. The selective A_2A_AR agonist 2-*p*-(2-carboxyethyl)phenethylamino-5′-*N*-ethylcarboxamidoadenosine hydrochloride (CGS21680), concentration-dependently induced cell proliferation in A375, A549, and MRMT1 cancer cells and the effect was potently antagonized by the A_2A_AR antagonist TP455, as well as by the reference A_2A_AR blocker 4-(2-[7-amino-2-(2-furyl)[1,2,4]triazolo[2,3-a][1,3,5]triazin-5-ylamino]ethyl)phenol (ZM241385). As for the signaling pathway recruited in this response we demonstrated that, by using the specific inhibitors of signal transduction pathways, the effect of A_2A_AR stimulation was induced through phospholipase C (PLC) and protein kinase C-delta (PKC-δ). In addition, we evaluated, through the AlphaScreen SureFire phospho(p) protein assay, the kinases enrolled by A_2A_AR to stimulate cell proliferation and we found the involvement of protein kinase B (AKT), extracellular regulated kinases (ERK1/2), and c-Jun N-terminal kinases (JNKs). Indeed, we demonstrated that the CGS21680 stimulatory effect on kinases was strongly reduced in the presence of the new potent compound TP455, as well as by ZM241385, confirming the role of the A_2A_AR. In conclusion, the A_2A_AR activation stimulates proliferation of A375, A549, and MRMT1 cancer cells and importantly TP455 reveals its capability to counteract this effect, suggesting selective A_2A_AR antagonists as potential new therapeutics.

## Introduction

Adenosine, a ubiquitous purine nucleoside, is considered as an important modulator of tissue function, increasing its concentrations under adverse metabolic conditions. Adenosine is produced in the extracellular space through ATP degradation operated by specific ectoenzymes, named apyrase (CD39) and 5′-nucleotidase (CD73) and exerts its effects by recruitment of four G-protein-coupled A_1_, A_2A_, A_2B_, and A_3_ adenosine receptors (ARs) (Borea et al., 2016). A_1_AR activation by interacting with Gi/Go proteins inhibits adenylyl cyclase (AC), regulates calcium and potassium channels, as well as phospholipase C (PLC). The A_2A_AR couples to Gs/Golf proteins to activate AC thus increasing cAMP levels. The A_2B_ receptor, by recruiting Gs/Gq protein raises AC and activates PLC. Finally, the A_3_AR interacts with Gi and Gq proteins inhibiting AC and stimulating PLC, respectively (Fredholm et al., 2011). In addition, in a cell type specific way, all adenosine receptors may also be linked to mitogen-activated protein kinases (MAPK), including extracellular signal-regulated kinase (ERK) 1/2, c-Jun-N-terminal kinase 1/2 (JNK1/2), and p38 MAPK kinase, crucial in the modulation of cell growth and death (Schulte and Fredholm, 2000). Indeed, an important role of adenosine in human cancerogenesis has been evidenced, as it regulates almost all the phases of cancer development including immunoescaping, cell proliferation, angiogenesis and metastasis, by recruiting different adenosine receptor subtypes (Antonioli et al., 2013a,b; Borea et al., 2017).

It is well established the relevance of immune cells in the fight against tumors and adenosine, that increases in hypoxic solid tumors, decreases the recognition of cancer cells by cytolytic T cells (Blay et al., 1997; Merighi et al., 2003; Muller-Haegele et al., 2014). Specifically, these cells are depressed by A_2A_ARs with the final result of an increase in hypoxic tumor cell survival and immunoescaping as demonstrated in A_2A_AR gene-deficient mice, having a much stronger antitumor immunity, rejection of established tumors and prolonged animal survival (Ohta et al., 2006; Sitkovsky et al., 2008; Young et al., 2016). Furthermore, A_2A_ARs promote wound healing and angiogenesis and are able to increase also melanoma and breast cancer cell proliferation (Merighi et al., 2002; Etique et al., 2009; Koszałka et al., 2016; Perez-Aso et al., 2016). All these data support the importance of A_2A_AR antagonists to combat tumor development.

**FIGURE 1:**
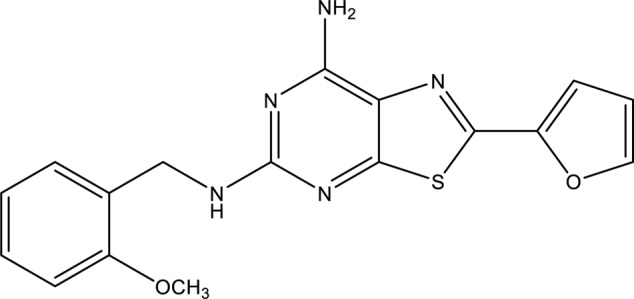
Chemical structure of the A_2A_AR antagonist/inverse agonist TP455 (2-(2-furanyl)-N^5^-(2-methoxybenzyl)[1,3]thiazolo[5,4-d]pyrimidine-5,7-diammine).

Recently, a series of novel blockers, having the thiazolo[5,4-d]pyrimidine nucleus, showing an unprecedented high affinity for the A_2A_AR and a behavior as antagonists and/or inverse agonists has been developed (Varano et al., 2016). With the availability of the novel compound 2-(2-furanyl)-N^5^-(2-methoxybenzyl)[1,3]thiazolo[5,4-d]pyrimidine-5,7-diammine (TP455) (**Figure 1**), the involvement of A_2A_ARs in human A375 melanoma, as well as in human A549 lung and rat MRMT1 breast carcinoma proliferation was evaluated. In addition, the signaling pathways triggered in the cancer cells of different origin and the antagonist effect of TP455 were investigated.

Overall our data indicate that the A_2A_AR activation stimulates proliferation of A375, A549, and MRMT1 cancer cells through ERK1/2, JNK1/2, and AKT downstream PLC and PKC-δ. Importantly, TP455 antagonizes this effect, adding a piece of evidence on the effects of selective A_2A_AR antagonists on cancer development thus supporting their role as potential new anticancer drugs.

## Materials and Methods

The A_2A_AR antagonist/inverse agonist TP455 was recently synthesized (compound 13 in Varano et al., 2016) and the chemical structure is shown in **Figure 1**. [^3^H]-ZM 241385 was from PerkinElmer (Milan, Italy). 4-(2-[7-Amino-2-(2-furyl) [1,2,4]triazolo[2,3-a][1,3,5]triazin-5-ylamino]ethyl)phenol (ZM241385) and 8-[4-[4-(4-Chlorophenzyl)piperazide-1-sulfonyl)phenyl]]-1-propylxanthine (PSB 603) were purchased from Tocris, Space Import–Export (Milan, Italy). 4- (4-Fluorophenyl)-2-(4-hydroxyphenyl)-5-(4-pyridyl)-1H-imidazole (SB202190) was purchased by Adipogen (Florence, Italy). D-3-Deoxy-2-*O*-methyl-myo-inositol 1-[(R)-2-methoxy-3-(octadecyloxy)propyl hydrogen phosphate] (SH5) and 1,9-Pyrazoloanthrone (SP600125) were from Enzo Life (Florence, Italy). 1-[6-[[(17β)-3-Methoxyestra-1,3,5(10)-trien-17-yl]amino]hexyl]-1H-pyrrole-2,5-dione (U73122) was from Cayman (Florence, Italy). 2-*p*-(2-Carboxyethyl)phenethylamino-5′-N-ethylcarboxamido-adenosine hydrochloride hydrate (CGS 21680), 5′-(*N*-Ethylcarboxamido)adenosine (NECA), 1-[6-[(3-Acetyl-2,4,6-trihydroxy-5-methylphenyl)methyl]-5,7-dihydroxy-2,2-dimethyl-2H-1-benzopyran-8-yl]-3-phenyl-2-propen-1-one (Rottlerin) and 2-(2-Furanyl)-7-[3-(4-methoxyphenyl)propyl]-7H-pyrazolo[4, 3-e][1,2,4]triazolo[1,5-c]pyrimidin-5-amine (SCH 442416) were purchased from Sigma (Milan, Italy). PKC-𝜀 translocation inhibitor peptide was purchased by Calbiochem (Milan, Italy). AlphaScreen SureFire phospho(p)ERK1/2(Thr202/Tyr204), p-AKT1/2/3 (pThr308) and p-JNK1/3 (pThr183/Tyr185) assay kits, AlphaScreen^®^ cAMP and DELFIA^®^ Cell Proliferation kit were from PerkinElmer (Milan, Italy). Unless otherwise noted, all other reagents were purchased from Sigma (Milan, Italy).

### Cell Culture Conditions

Tumoral cell lines A375 (human skin malignant melanoma), A549 (adenocarcinomic human alveolar basal epithelial cells) and rat cell line MRMT-1 (rat breast carcinoma cells) were purchased from ATCC and were grown adherently at 37°C in 5% CO_2_/95% air. A375 and A549 were maintained in DMEM high glucose medium containing 10% fetal calf serum, penicillin (100 U/mL) and streptomycin (100 mg/mL) and MRMT-1 were grown in RPMI 1640 medium containing 10% fetal calf serum, penicillin (100 U/mL), streptomycin (100 mg/mL), and L-glutamine (2 mM). Adenosine receptor agonists and antagonists and inhibitors of kinases, were made up in dimethyl sulfoxide solution (DMSO) and then diluted in cell culture medium (0.1 max 0.2% of DMSO). An equal amount of DMSO was used in control cells (CTR).

### Membrane Preparation

For membrane preparation the culture medium was removed. The cells were washed with PBS and scraped off 90 mm diameter petri dishes in ice-cold hypotonic buffer (5 mM Tris–HCl, 2 mM EDTA, pH 7.4) (Gessi et al., 2007). The cell suspension was homogenized with Polytron and the homogenate was spun for 10 min at 1000 g. The supernatant was then centrifuged for 30 min at 100,000*g*. The membrane pellet was resuspended in 50 mM Tris HCl buffer pH 7.4, 10 mM MgCl_2_. The protein concentration was determined according to a Bio-Rad method with BSA as a standard reference (Bradford, 1976). Then the suspension was frozen at -80°C.

### Saturation and Competition Binding Experiments

Saturation binding experiments on A549, A375, and MRMT-1 cell membranes were performed by using [^3^H]-ZM 241385 at different concentrations (0.1–30 nM), incubated with 100 μg of protein per assay of membrane suspension, for 1 h at 4°C. Competition experiments of [^3^H]-ZM 241385 were performed in duplicate in test tubes containing the buffer, the membranes and different concentrations of A_2A_ARs agonists and antagonists. Non-specific binding was defined as the binding in the presence of 1 μM ZM 241385 and was <32% of the total binding. At the end of the incubation, bound and free radioactivity were separated by filtering, in a Brandel cell harvester, the assay mixture through Whatman GF/B glass-fiber filters. The filter bound radioactivity was counted in a liquid Scintillation Counter Tri Carb Packard 2500 TR (Perkin-Elmer Life and Analytical Sciences, Boston, MA, United States).

### AlphaScreen SureFire Assays

AlphaScreen SureFire phospho(p)ERK1/2(Thr202/Tyr204), pJNK1/3(pThr183/Tyr185), and p-AKT1/2/3 (pThr308) assay kits (Perkin Elmer, Milan, Italy) were utilized. Upon kinase phosphorylation and excitation at 680 nm, fluorescent signals at 615 nm are emitted. Cells were seeded in 100 μl culture medium into 96-well plates (30,000/well), and incubated at 37°C for 24 h. Cells were pretreated with various inhibitors and TP455 for 30 min. Then, receptors were maximally stimulated using 100 nM CGS 21680 and incubated for 5 min (ERK1/2, JNK1/2-MAPK) or 30 min (AKT) at 37°C. After agonist removal, lysis buffer was added, then donor and acceptor beads linked to specific anti-*p*-kinase- and anti-kinase-antibodies were dispensed, according to manufacturer instructions. Finally, fluorescent signals were detected through an Ensight Perkin Elmer-multimode plate reader (Perkin Elmer, Milan, Italy). Data were normalized to fold activation above basal *p*-kinase levels (=100). For inhibitor graphs, raw data were transformed into percentages relative to controls (basal level = 100%) in order to merge data from several experiments.

### DELFIA Cell Proliferation Kit

The DELFIA assay was performed to determine cell proliferation according to the manufacturer’s protocol from PerkinElmer (Milan, Italy). The assay is a time-resolved fluoroimmunoassay based on the incorporation of BrdU into newly synthesized DNA strands of proliferating cells cultured in microliter plates. Incorporated BrdU is detected using a europium labeled monoclonal antibody and the fluorescence measured is proportional to the DNA synthesis in the cell population of each well. A375, A549, and MRMT-1 cells were cultured over night at 1000 cells/well in a 96-well plate (at a final volume of 100 μl per well), agonists and antagonists were added and the cells were incubated for 30′ before addition of the BrdU-Labeling solution 10 μl/well. The cells were then cultured for 24, 48, or 72 h. At the end of the incubation period, cells were fixed (fix solution 100 μl/well), added with 100 μl/well of Anti-BrdU-Eu (0.5 μg/ml) and incubated for 120 min at room temperature. After four washes, 200 μl of DELFIA Inducer were added at room temperature for 15 min and the Eu-fluorescence was detected through an Ensight Perkin Elmer-multimode plate reader (Perkin Elmer, Milan, Italy). Two kinds of controls were performed: the blank where no cells were added to the well but only culture medium and the background where no BrdU was added to the wells.

### Statistical Analysis

For saturation binding experiments, determination of receptor affinity (K_D_) and receptor density (BMAX) was performed using the non-linear least-squares curve fitting program LIGAND (Munson and Rodbard, 1980). LIGAND was also used to determine inhibitory binding constant (Ki) values from the competition binding experiments. All values in the figures and text are expressed as mean ± standard error (SE) of three independent experiments. Data sets were examined by one-way analysis of variance (ANOVA) and Dunnett’s test (when required). ^∗^*P* < 0.05 was considered significant.

## Results

### Saturation Studies

**FIGURE 2:**
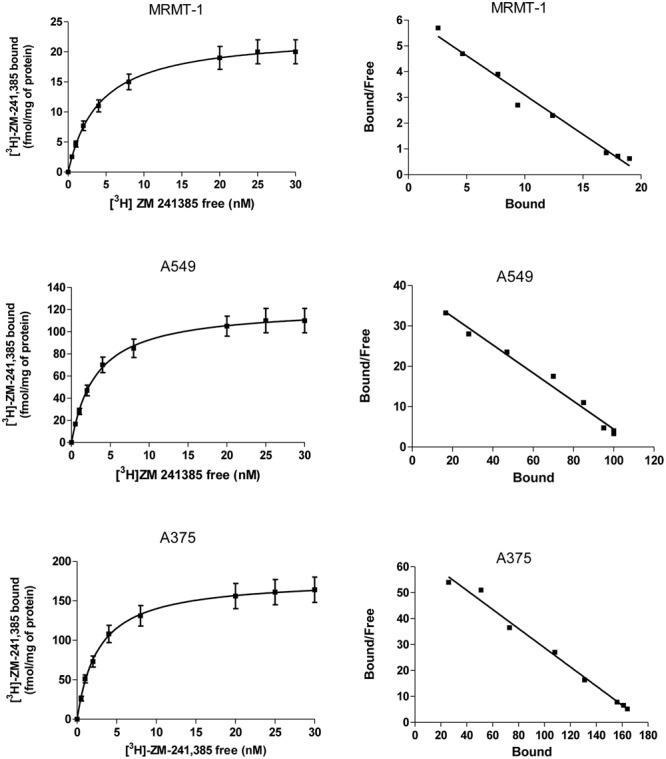
Saturation curves of [^3^H]-ZM 241385 binding in A375, A549, and MRMT-1 cell lines. Experiments were performed as described in Section “Materials and Methods”. Values are the means and vertical lines SE of the mean of four separate experiments performed in triplicate. The Scatchard plots of the same data are shown.

The expression of A_2A_ARs in A375, A549, and MRMT-1 cells was determined performing saturation binding experiments with [^3^H]-ZM 241385. The saturation curves of [^3^H]-ZM 241385 binding, reported in **Figure 2**, show a *K*_D_ value of 2.75 ± 0.25, 2.98 ± 0.31 nM, 2.17 ± 0.18 nM, and a *B*_max_ of 178 ± 20, 110 ± 9, 21 ± 4 fmol mg^-1^of protein in A375, A549, and MRMT-1 cells, respectively. The Scatchard plots in the insert are linear failing to show a significantly better fit to a two-site than to a one-site binding model, demonstrating that only one class of high affinity binding sites is present in our experimental conditions (Munson and Rodbard, 1980).

### Competition Experiments

The affinity values of the examined ligands obtained in [^3^H]-ZM 241385 competition binding experiments performed in A375, A549, and MRMT-1 cell membranes were determined, as shown in **Table 1**. The order of potency of the agonists was as follows: NECA > CGS 21680 > R-PIA = Cl-IB-MECA. The order of potency of the antagonists was as follows: TP455 > CGS15943 > ZM241385 > SCH58261. Specifically, the novel compound TP455 revealed a very good affinity for rat and human A_2A_ARs with Ki value in the picomolar range in all the three cell lines investigated.

**Table 1:** Inhibition of [^3^H]ZM241385 binding (Ki nM) by adenosine receptor agonists and antagonists in human A375 melanoma, A549 lung carcinoma, and rat MRMT-1 breast carcinoma membranes.

Compounds	A375 Ki (nM)	A549 Ki (nM)	MRMT-1 Ki (nM)
**Agonists**			
CGS21680	15 1	16 2	14 1
NECA	6.65 0.52	12 1	9.33 0.95
Cl-IB-MECA	694 75	710 89	500 64
R-PIA	725 78	670 75	423 52
**Antagonists**			
SCH58261	2.82 0.32	3.15 0.34	3.26 0.43
ZM241385	2.23 0.26	2.33 0.24	2.47 0.23
CGS15943	1.45 0.18	1.66 0.20	2.15 0.34
TP455	0.0058 0.0002	0.0053 0.0003	0.0061 0.0003

*The data are expressed as mean ± SE.*

### Increase in Cell Proliferation Induced by CGS 21680 in Cancer Cell Lines

**FIGURE 3:**
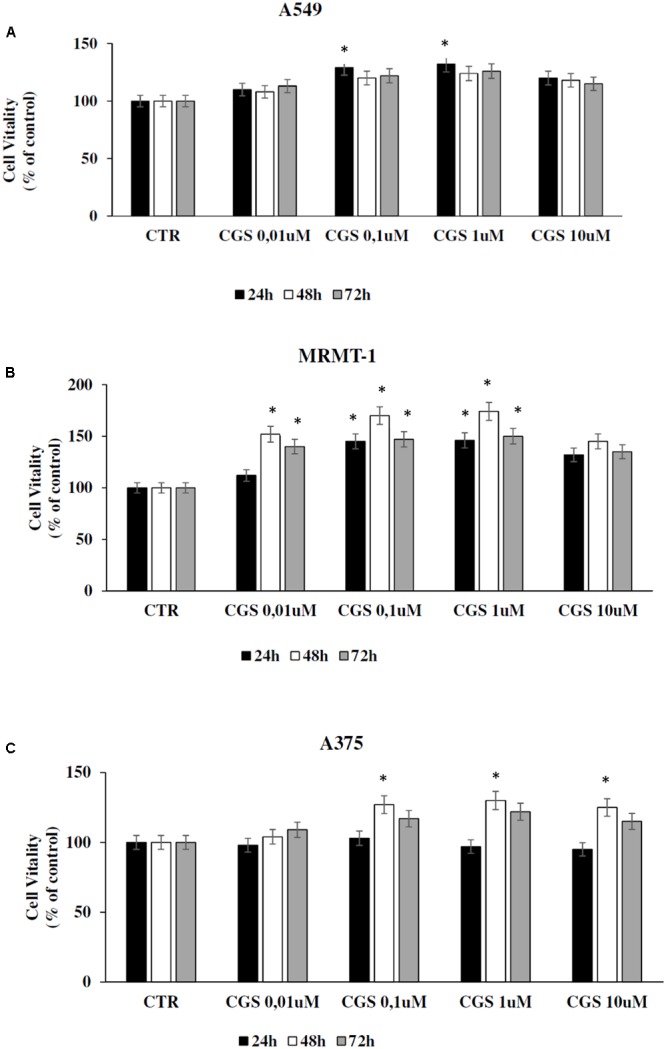
Effect of CGS 21680 on cell proliferation in A549 **(A)**, MRMT-1 **(B)**, and A375 **(C)** cell lines. Cells were incubated in the presence of 0.01–10 μM CGS 21680 for 24, 48, and 72 h and cell proliferation was evaluated by DELFIA Cell Proliferation Kit. Solutions were made up in DMSO and then diluted in cell culture medium (0.1 max 0.2% of DMSO). An equal amount of DMSO was used in control cells (CTR). *^∗^P* < 0.01 compared with CTR. Means ± SE values from four experiments are shown. Analysis was by one way ANOVA, followed by Dunnett’s test.

The effect of A_2A_ARs activation on tumor cell proliferation was evaluated in A549, MRMT-1, and A375 cancer cells. Specifically, the A_2A_ARs selective agonist CGS21680 (10–100 nM) was applied to cancer cells for 24, 48, and 72 h of incubation before assessing proliferation. The results show that in A549 cells CGS 21680 slightly increased cell proliferation only when used at the concentration of 100 nM for 24 h and its effect was not more present after 48 and 72 h (**Figure 3A**). As for MRMT-1 cells the A_2A_ARs agonist at 10 nM raised cell vitality after 48 and 72 h of treatment, while a significant stimulation was observed with CGS 21680 100 nM at all the time points investigated (**Figure 3B**). In A375 cells the stimulatory effect of cell proliferation A_2A_ARs-dependent was revealed only at 48 h of treatment with CGS21680 100 nM (**Figure 3C**).

### Antagonism of CGS 21680-Induced Cell Proliferation in Cancer Cell Lines by the New A_2A_ARs Selective Antagonist TP455

**FIGURE 4:**
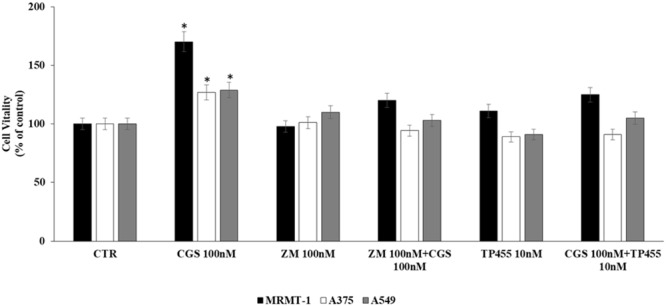
Effect of 100 nM CGS 21680 on A375 and MRMT-1 cell viability (48 h) as well as in A549 (24 h) and antagonism by 100 nM ZM 241385 and 10 nM TP455 by DELFIA Cell Proliferation Kit. Solutions were made up in DMSO and then diluted in cell culture medium (0.1 max 0.2% of DMSO). An equal amount of DMSO was used in control cells (CTR) incubated for 24 h in case of A549 and 48 h in case of A375 and MRMT-1. ^∗^*P* < 0.01 compared with CTR. Means ± SE values from four experiments are shown. Analysis was by one way ANOVA, followed by Dunnett’s test.

In order to verify that the increase of cell proliferation induced by CGS21680 was mediated through A_2A_ARs stimulation we antagonized its effect by using the standard antagonist ZM 241385. MRMT-1, A375 as well as A549 cells were pretreated for 30 min with 100 nM ZM 241385 before stimulation with 100 nM CGS21680 for 48 h with exception of A549 tested after 24 h. As shown in **Figure 4** this compound was able to completely block the agonist effect in all the cell lines studied, confirming the involvement of A_2A_ARs in cancer cell proliferation. Therefore, the ability of the new selective and high affine A_2A_ARs compound TP455 to revert cell proliferation induced by CGS21680 was investigated. Our results show that the increase in cell vitality CGS21680-dependent was antagonized by addition of 10 nM TP455 in all cancer cells, suggesting that this novel derivative behaves as an A_2A_ARs antagonist (**Figure 4**). When tested alone 10 nM TP455 and 100 nM ZM 241385 did not alter cell proliferation, showing a behavior of pure A_2A_AR antagonists (**Figure 4**).

### Signaling Pathways Involved in Cell Proliferation Induced by CGS 21680 in Cancer Cell Lines

**FIGURE 5:**
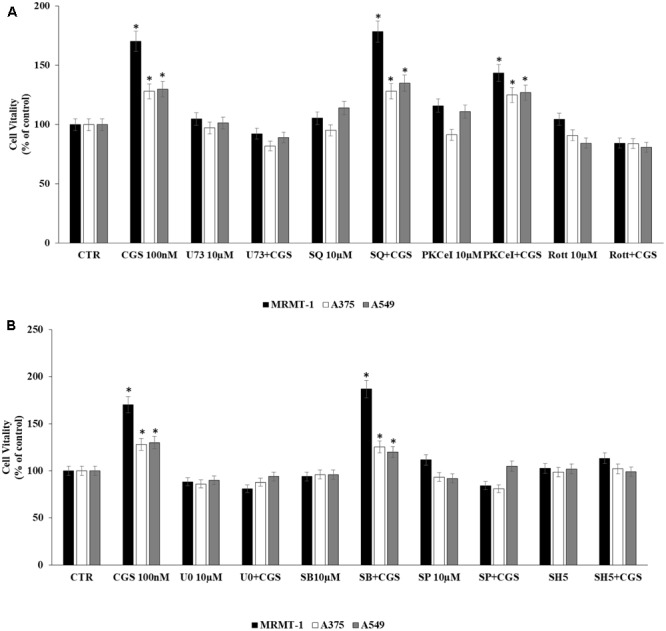
Signaling pathways involved in cell proliferation induced by CGS 21680 in cancer cell lines. Effect of 100 nM CGS 21680 on A375, MRMT cell viability (48 h) as well as in A549 (24 h) and inhibition by 10 μM U73, SQ, PKC𝜀-I, and Rott **(A)**, 10 μM SB202190 (SB), U0126 (U0), SP600125 (SP), and SH5 **(B)** by DELFIA Cell Proliferation Kit. Solutions were made up in DMSO and then diluted in cell culture medium (0.1 max 0.2% of DMSO). An equal amount of DMSO was used in control cells (CTR) incubated for 24 h in case of A549 and 48 h in case of A375 and MRMT-1. ^∗^*P* < 0.01 compared with CTR. Means ± SE values from four experiments are shown. Analysis was by one way ANOVA, followed by Dunnett’s test.

The involvement of PLC, AC, PKC𝜀, and PKCδ in the increase of cell proliferation due to A_2A_AR activation was investigated. Cells were incubated with U73122 (U73), SQ22,536 (SQ), PKC𝜀-translocation inhibitor peptide (PKC𝜀-I), and rottlerin (Rott) as inhibitors of PLC, AC, PKC𝜀, and PKCδ, respectively. MRMT-1, A375 as well as A549 cells were pretreated for 30 min with 10 μM inhibitors before stimulation with 100 nM CGS21680 for 48 h with exception of A549 tested after 24 h. All inhibitors alone did not significantly affect cell proliferation (**Figure 5A**). As shown in **Figure 5A** blockers of PLC and PKCδ were able to antagonize the stimulatory effect of 100 nM CGS21680, suggesting the involvement of these enzymes in the A_2A_AR agonist effect, while inhibitors of AC and PKC𝜀 did not block the agonist effect.

In addition, to evaluate MAPK and AKT pathways involvement in A_2A_AR-mediated cell proliferation, cells were pretreated for 30 min with 10 μM U0126, SB202190, SP600125, and SH5, inhibitors of ERK1/2, p38, JNK1/2 MAPK kinases and AKT, respectively, before exposure to 100 nM CGS 21680 for 48 h with exception of A549 tested after 24 h. All inhibitors alone did not significantly affect cell proliferation (**Figure 5B**). As shown in **Figure 5B**, U0126, SP600125, and SH5 strongly reduced the effect of CGS21680 on cell proliferation, whilst SB202190 did not. These results suggest the involvement of ERK1/2, JNK1/2 MAPK kinases, and AKT in the increase of cell proliferation mediated by A_2A_AR activation.

### Effect of CGS 21680 on ERK1/2, JNK MAPK Kinase, and AKT Phosphorylation

**FIGURE 6:**
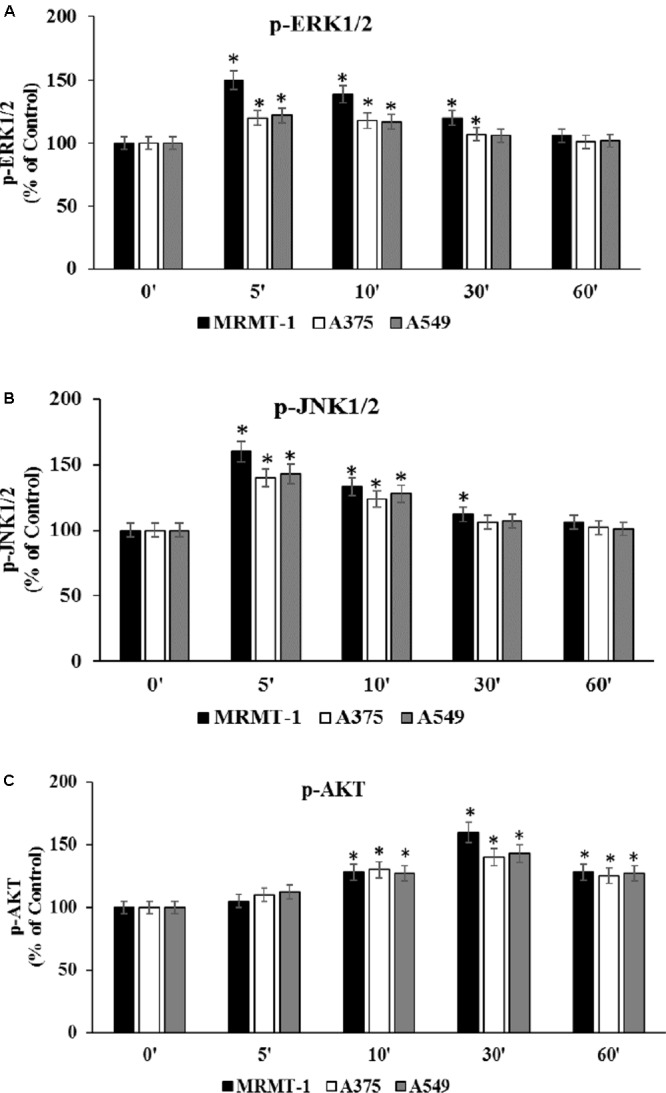
Effect of CGS 21680 on pERK1/2, pJNK1/2 MAPK kinase, and pAKT activation. Cells were incubated in the presence of 100 nM CGS 21680 for 0, 5, 10, 30, and 60 min and pERK1/2 **(A)**, pJNK1/2 **(B)**, and pAKT **(C)** protein levels were evaluated by AlphaScreen SureFire pMAPK assays in MRMT-1, A375, and A549 cells. Solutions were made up in DMSO and then diluted in cell culture medium (0.1 max 0.2% of DMSO). An equal amount of DMSO was used in control cells (CTR). ^∗^*P* < 0.01 compared with time 0. Means ± SE values from four experiments are shown. Analysis was by one-way ANOVA followed by Dunnett’s test.

To confirm the role of ERK1/2, JNK1/2 MAPK kinases, and AKT in the increase of cell proliferation mediated by A_2A_AR activation, kinases phosphorylation was assessed at 0–60 min in the absence and in the presence of 100 nM CGS 21680, as stimulator of A_2A_ARs in MRMT-1, A375 and A549 cells (**Figures 6A–C**, respectively). ERK1/2 and JNK1/2 phosphorylation reached a maximal effect after 5 min of A_2A_AR stimulation and disappeared at 60 min, while AKT phosphorylation started to increase after 10 min of treatment and decreased after 60 min. Furthermore, ERK1/2, JNK, and AKT phosphorylation were higher in MRMT-1 compared to A375 and A549 cells (**Figures 6A–C**, respectively).

### Antagonism of CGS 21680-Induced ERK1/2, JNK1/2 MAPK Kinase and AKT Phosphorylation by the New A_2A_ARs Selective Antagonist TP455

**FIGURE 7:**
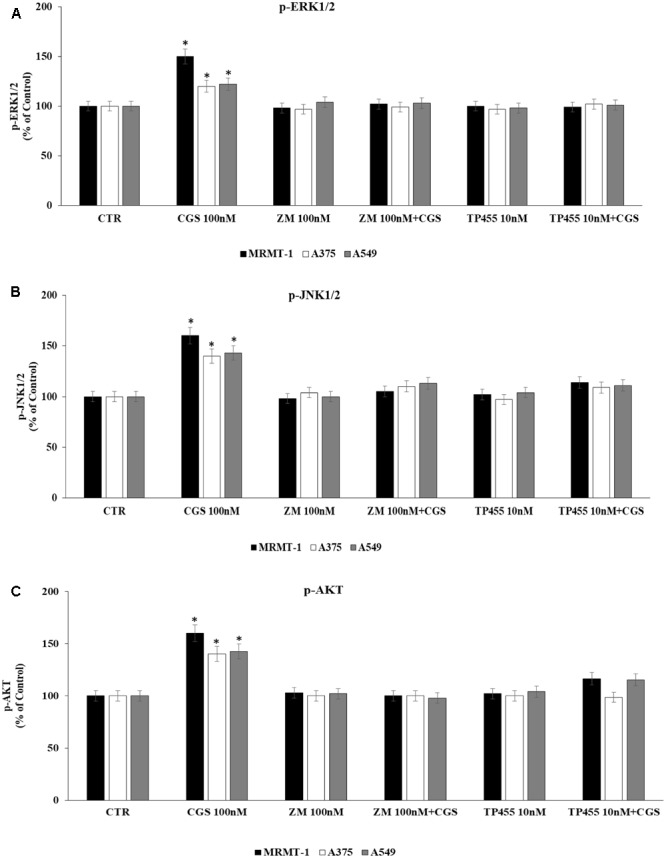
Effect of antagonists on CGS 21680-dependent pERK1/2, pJNK MAPK kinase, and pAKT activation. MRMT-1, A375, and A549 cells in the absence or in the presence of 100 nM ZM 241385 and 10 nM TP455 were exposed to 100 nM CGS for 5 min or for 30 min and pERK1/2 **(A)**, pJNK **(B)**, and pAKT **(C)** protein levels were evaluated by AlphaScreen SureFire pMAPK assays respectively. Solutions were made up in DMSO and then diluted in cell culture medium (0.1 max 0.2% of DMSO). An equal amount of DMSO was used in control cells (CTR). ^∗^*P* < 0.01 compared with CGS 21680 100 nM. Means ± SE values from four experiments are shown. Analysis was by one-way ANOVA followed by Dunnett’s test.

To evaluate whether stimulation of ERK1/2, JNK1/2, and AKT phosphorylation CGS21680-dependent was induced through A_2A_AR activation we antagonized it by using the standard antagonist ZM 241385. MRMT-1, A375, and A549 cells were pretreated for 30 min with 100 nM antagonist before stimulation with 100 nM CGS21680 for 5 (ERK1/2, JNK1/2) or 10 min (AKT) to assess kinases phosphorylation. As shown in **Figures 7A–C** the effect of CGS 21680 on ERK1/2, JNK1/2, and AKT phosphorylation, respectively, was completely reverted by ZM 241385. In addition the ability of the A_2A_AR antagonist TP455 to block kinases phosphorylation induced by CGS21680 was studied. Our results show that this effect was potently inhibited by pre-treatment for 30 min with 10 nM TP455 in MRMT-1, A375, and A549 cells (**Figures 7A–C**).

### Characterization of ERK1/2, JNK1/2 MAPK Kinase, and AKT Signaling Cascade Triggered by CGS 21680 in Cancer Cell Lines

**FIGURE 8:**
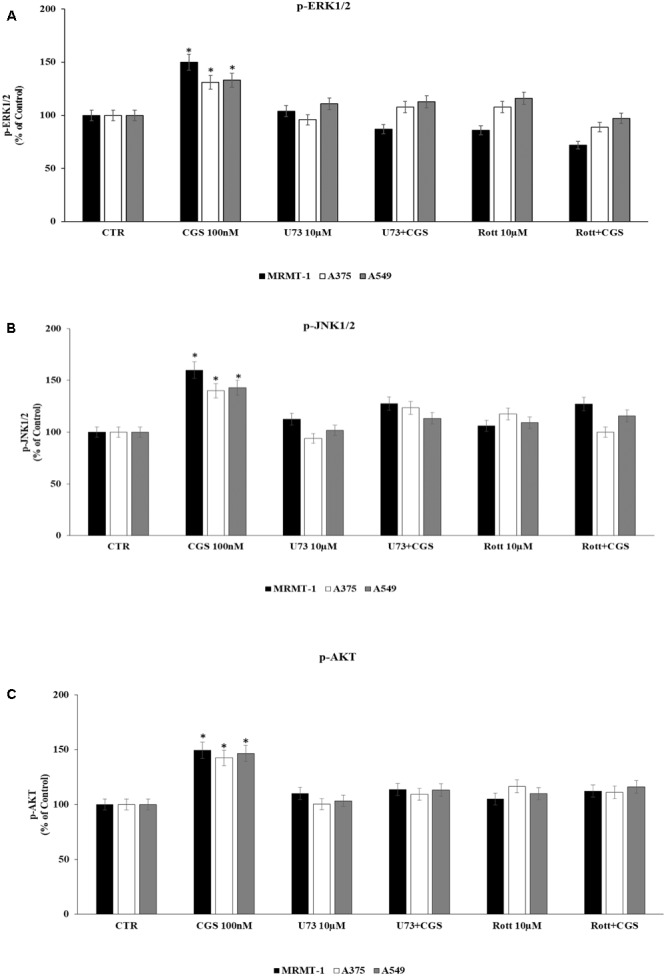
Effect of 100 nM CGS 21680 on pERK1/2 **(A)**, pJNK1/2 **(B)**, and pAKT **(C)** protein levels in MRMT-1, A375 and A549 cells and antagonism by 10 μM U73122 (U73) and 10 μM rottlerin (Rott) evaluated by AlphaScreen SureFire pMAPK assays. Solutions were made up in DMSO and then diluted in cell culture medium (0.1 max 0.2% of DMSO). An equal amount of DMSO was used in control cells (CTR). ^∗^*P* < 0.01 compared with CTR. Means ± SE values from four experiments are shown. Analysis was by one-way ANOVA followed by Dunnett’s test.

Finally, in order to deeply investigate the signaling cascade triggered by A_2A_AR activation in the increase of ERK1/2, JNK1/2, and AKT phosphorylation, MRMT-1, A375, and A549 cells were incubated with PLC and PKCδ inhibitors before the exposure to 100 nM CGS 21680. Then ERK1/2, JNK1/2, and AKT phosphorylation status was examined. As reported in **Figure 8** phosphorylation of ERK1/2, JNK1/2, and AKT mediated by CGS21680 was strongly reduced in the presence of 10 μM U73 and Rott suggesting that PLC and PKCδ were upstream of ERK1/2, JNK1/2 and AKT (**Figures 8A–C**, respectively).

## Discussion

Several data in literature support an important role of adenosine in tumor development. Indeed, adenosine may act as both an anti- or pro-tumoral endogenous nucleoside depending on which adenosine receptor subtype is recruited (Borea et al., 2016, 2017). Specifically, as for the anti-tumoral effect, the main receptor subtype involved results the A_3_AR for which the successful data obtained in preclinical studies lead to the development of A_3_AR selective agonists, now under evaluation in clinical studies for the treatment of hepatocellular carcinoma (Stemmer et al., 2013; Jacobson et al., 2017). Instead, as for the protumoral effect, it is well established the enrollment of A_2A_AR subtype in immune depression of anticancer response, in angiogenesis stimulation as well as in promotion of cancer cell migration, suggesting a role of A_2A_ARs antagonists in the fight against cancer (Antonioli et al., 2013a,b, 2016). In addition, A_2A_ARs have been suggested as regulators of cancer cell proliferation in melanoma and breast cancer, but the signaling pathways have not been deeply depicted (Merighi et al., 2002; Etique et al., 2009; Mediavilla-Varela et al., 2013).

In this study, we investigated the effect of A_2A_AR activation in the modulation of cell proliferation in A375 melanoma, A549 lung and MRMT-1 breast carcinoma, the mechanisms involved and the efficacy of a novel antagonist to counteract A_2A_AR effects on cell growth.

Our results show that A_2A_ARs are expressed in all the three cancer cell lines investigated, with the following order of expression: A375 > A549 > MRMT-1 as evaluated through receptor binding experiments. In order to investigate the role of A_2A_ARs in cancer growth we evaluated cancer cell proliferation, following the treatment with a selective A_2A_AR agonist, and we found an increase of it, with a most significant effect in MRMT-1 cells. The mechanism involved does not affect AC or PKC-𝜀 pathways but recruited PLC and PKC-δ stimulation. In addition, cell proliferation mediated by A_2A_ARs was blocked by selective ERK1/2, JNK1/2, and AKT but not p38 inhibitors. Accordingly, phosphorylation of ERK1/2, JNK1/2, and AKT was significantly increased by A_2A_ARs. It is interesting to note that ERK1/2, JNK1/2, and AKT activation by A_2A_ARs depends on PLC and PKC- δ stimulation, as demonstrated through the block of kinases phosphorylation following treatment with PLC and PKC- δ inhibitors. These data suggest that PLC and PKC- δ are upstream ERK1/2, JNK1/2, and AKT in the signaling proliferative pathway induced by A_2A_ARs.

**FIGURE 9:**
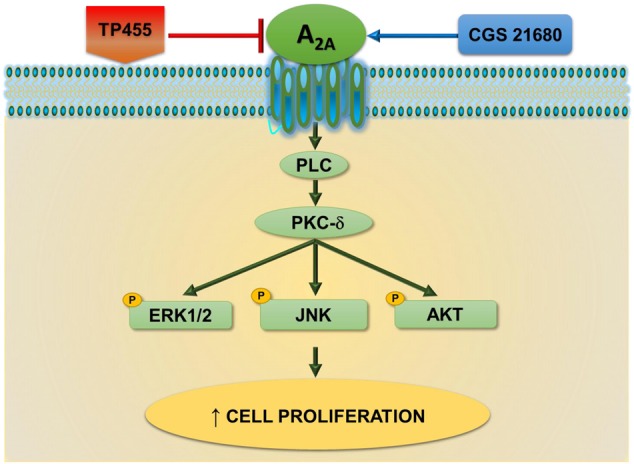
A_2A_ adenosine receptors-triggered signal transduction cascade in cancer cells. CGS21680 agonist by activating A_2A_ARs increases cell proliferation through a pathway dependent on PLC, PKCδ, pERK1/2, pJNK, and pAKT signaling. This effect is potently antagonized by the newly synthesized derivative TP455.

These data are in agreement with other studies where A_2A_AR activation was associated to an increase in cell growth such as A375 melanoma, MCF-7 breast and A549 lung carcinoma (Gessi et al., 2011). Specifically, in A375 cells the association of A_2A_AR mediated cell proliferation with ERK1/2 has been already observed (Merighi et al., 2002). However, in A549 cell line, a direct increasing apoptotic effect, due to A_2A_AR antagonist treatment was observed, even though the high concentration used in the reported study does not exclude the possible interaction with other receptors (Mediavilla-Varela et al., 2013). Therefore, the novelty of this work is to depict a novel signaling cascade involving the classical kinases ERK1/2, JNK1/2, and AKT downstream A_2A_ARs, PLC, and PKC- δ, linked in the tumorigenic processes (**Figure 9**).

All the effects observed in this study, activated by the A_2A_AR agonist, were reversed by the standard A_2A_AR antagonist ZM241385, confirming the role of A_2A_ARs in the signaling investigated. Importantly, the behavior of the novel compound TP455 similar to ZM241385, able to potently block A_2A_AR- induced cancer cell proliferation and kinase phosphorylation, indicates a role for it as a novel potent A_2A_AR antagonist. Interestingly, this class of compounds is already under clinical development due to its anti-Parkinson therapeutic effects and is well known to be safe and well tolerated (Preti et al., 2015; Jazayeri et al., 2017).

Overall our data help to implement the knowledge concerning the signaling of A_2A_ARs in cancer. However, to support the relevance of these receptors as novel targets in the therapy against cancer and the development of potent and selective antagonists, it will be important to evaluate whether A_2A_ARs are also involved in normal cell proliferation and the signaling pathway enrolled.

## Author Contributions

SG and SM developed the original idea, designed the experiments and elaborated data. SB and EB performed experiments and prepared figures, KV and FaV elaborated data, FlV, DC, and VC edited and reviewed the final version of the article, PAB supervised the study. All listed authors contributed to article writing.

## Conflict of Interest Statement

The authors declare that the research was conducted in the absence of any commercial or financial relationships that could be construed as a potential conflict of interest.
